# Adipokines, and not vitamin D, associate with antibody immune responses following dual BNT162b2 vaccination within individuals younger than 60 years

**DOI:** 10.3389/fimmu.2022.1000006

**Published:** 2022-09-02

**Authors:** Mariana Pavel-Tanasa, Daniela Constantinescu, Corina Maria Cianga, Ecaterina Anisie, Ana Irina Mereuta, Cristina Gabriela Tuchilus, Petru Cianga

**Affiliations:** ^1^ Department of Immunology, Faculty of Medicine, Grigore T. Popa University of Medicine and Pharmacy, Iasi, Romania; ^2^ Laboratory of Immunology, St. Spiridon County Clinical Emergency Hospital Iasi, Iasi, Romania; ^3^ Medical Analysis Laboratory, St. Spiridon County Clinical Emergency Hospital Iasi, Iasi, Romania; ^4^ Department of Preventive Medicine and Interdisciplinarity (Microbiology), Faculty of Medicine, Grigore T. Popa University of Medicine and Pharmacy, Iasi, Romania

**Keywords:** BNT162b2 vaccination, SARS-CoV-2, antibody immune responses, adiponectin, MCP-1, PAI-1, age

## Abstract

Severe acute respiratory syndrome coronavirus-2 (SARS-CoV-2) led to a global health outbreak known as the COVID-19 pandemic which has been lasting since March 2020. Vaccine became accessible to people only at the beginning of 2021 which greatly helped reducing the mortality rate and severity of COVID-19 infection afterwards. The efficacy of vaccines was not fully known and studies documenting the immune responses following vaccination are continuing to emerge. Recent evidence indicate that natural infection prior vaccination may improve the antibody and cellular immune responses, while little is known about the factors influencing those processes. Here we investigated the antibody responses following BNT162b2 vaccination in relation to previous-infection status and age, and searched for possible biomarkers associated with the observed changes in immune responses. We found that the previous-infection status caused at least 8-times increase in the antibody titres, effect that was weaker in people over 60 years old and unaltered by the vitamin D serum levels. Furthermore, we identified adiponectin to positively associate with antibody responses and negatively correlate with pro-inflammatory molecules (MCP-1, factor D, CRP, PAI-1), especially in previously-infected individuals.

## Introduction

During the current COVID-19 pandemic, two questions have become central for settling the conditions required for an efficient immune protection against disease. Firstly, what is the durability of humoral immune responses after vaccination in infection-naïve or previously-infected individuals, and whether (or when) a vaccination boost is required for each of those, especially in the context of new up-coming variants? Of importance, there is increasing evidence that previously-infected individuals might have amplified antibody responses following vaccination that can be detected up to almost one year (1–3). Secondly, how does age and underlying comorbidities influence the humoral immune responses and the durability of neutralizing antibody titres? As such, a recent study performed in the United Kingdom (UK) has identified reduced humoral and cellular responses in people aged 65 years or older not previously exposed to natural infection, when compared to younger subjects (4). However, more studies are required to understand how age can impact on the vaccine efficacy in other countries and nations.

Many observational studies have augmented the evidence that deficient vitamin D serum levels are correlated with higher rate of incidence or severity of COVID-19 (5–7). Additional data have even suggested that supplementation with vitamin D could be critical in mitigating the COVID-19 progression to lessen its severity (8). Therefore, various recent clinical trials also investigate whether supplementation with vitamin D could optimize the efficacy of COVID-19 vaccines (reviewed in (9)). Interestingly, the new emerging data are showing little association between vitamin D status and antibody responses following vaccination (10, 11), suggesting that more studies are required for establishing the exact role of vitamin D in modulating the efficacy of severe acute respiratory syndrome coronavirus-2 (SARS-CoV-2) vaccines.

Another factor influencing the progression and mortality rate of COVID-19 infection was represented by the adipose tissue dysfunction. It is well established that adipose tissue is a major source of adipokines (adiponectin, leptin) and pro-inflammatory mediators (monocyte chemoattractant protein-1 (MCP-1), tumor necrosis factor (TNF-α), factor D, IL-6), which mitigate a long-lasting low-grade inflammation and prothrombotic conditions that are exacerbated in the context of SARS-CoV-2 infection, predisposing to cardio-respiratory failure (reviewed in (12, 13)). Indeed, adiponectin circulating levels were shown to be significantly reduced in patients with COVID-19 respiratory failure in multiple studies (14–16). Moreover, leptin, which serum levels largely depend on the total adipose mass, also provides a high impact on the immune system, as it favors monocytes/macrophages activation, pro-inflammatory cytokines release and a predominant Th-1 response, the three hallmarks of immune responses noticed in critical COVID-19 patients (16, 17).

Based on these up-listed considerations, we wondered what the antibody responses following dual BNT162b2 vaccination are in younger and older individuals from our population in relation to previous-infection status, and reveal, if any, the associations between the magnitude of RBD (Receptor-Binding Domain)-specific antibody titres and circulating levels of vitamin D and adipokines. Here, we have identified that people over 60 years show lower antibody responses compared to the younger counterparts, irrespective of the infection status. Additionally, we show that vitamin D has a limited association with the amplitude of anti-RBD responses and only in younger infection-naive individuals (not previously exposed to natural infection). Importantly, previously-infected individuals showed higher extent of antibody titres, which were associated with higher levels of circulating adiponectin and lower concentrations of pro-inflammatory biomarkers (MCP-1, factor D, C-reactive protein (CRP), plasminogen activator inhibitor 1 (PAI-1)), but only in people younger than 60 years.

## Materials and methods

### Study participants and serum collection

Blood samples were collected at St. Spiridon County Clinical Emergency Hospital (Iasi, Romania) between April 2021 and August 2021 from presumably healthy individuals following dual BNT162b2 (BioNTech-Pfizer) vaccination (21 days interval between the two doses) at a mean of 67 days (SEM 1.88, IQR 47-91) after the second dose. The samples were next stratified according to previous exposure to natural SARS-CoV-2 infection based on anamnestic data. The vaccination for previously infected individuals was performed at least 90 days after infection, as recommended by our national authorities. This study has been reviewed and approved by the institutional ethics committees (St. Spiridon County Clinical Emergency Hospital of Iasi) and informed consent was obtained from participants in this study. More precisely, 192 participants agreed for antibody testing, of which 122 for both vitamin D (25OH) and adipokine assessment, and 14 only for vitamin D levels detection. The information related to age and gender were included in a database together with a unique identifier, in order to keep the sample’s identity unknown to the researcher. Around 9% of the subjects were obese and 1/5 of them were older than 60 years, and none of the participants was recorded with autoimmune diseases in our hospital database.

None of the subjects had vitamin D supplementation before the blood sampling. Blood samples were collected in vacutainers with no anticoagulant and processed within 6 hours of receipt at the Laboratory of Immunology. More precisely, blood was spun at 2000 G for 5 min, and the serum was separated and aliquoted for storage at -80°C until further analysis.

### Sample processing for assessing the antibody responses and vitamin D levels

After thawing, the samples were centrifuged at 2000 G for 5 min. The assessment of antibody response against the spike-RBD (Receptor-Binding Domain) region of SARS-CoV-2 was performed using an electro-chemiluminescent microparticle immunoassay on cobas^®^ automatic platforms. The limit of detection for our assay was 0.8 U/mL, and samples with values > 0.8 were classified as antibody-positive. For the detection of total vitamin D (25-Hydroxyvitamin D) levels, an electrochemiluminescence binding assay was also used on cobas^®^ automatic platforms. The measuring range was 3.0-70.0 ng/mL (or 7.50‐175 nmol/L), with a functional sensitivity of 4.01 ng/mL.

### Quantification of adipokines and other cytokines

The concentration of various adipose tissue-related biomarkers was performed using a human obesity custom premixed kit from R&D systems and performed on a Luminex 100/200 platform. The samples were diluted 1:4 before being processed. The biomarkers included in the study were: monocyte chemoattractant protein-1 (MCP-1/CCL2), c-reactive protein (CRP), factor D, plasminogen activator inhibitor 1 (PAI-1/SERPINE1), interleukins 6 (IL-6) and 10 (IL-10), adiponectin, leptin, resistin, and tumor necrosis factor (TNF-α). Briefly, 50 μl of microparticle mixture were added to each well of the microplate and 50 μl of standards and samples were added on top and left for a 2 hours incubation at room temperature on a horizontal orbital microplate shaker set at 500 rpm. Following a washing procedure, 50 μl of diluted biotin antibody cocktail were added to each well and the plate was incubated for 1 hour at room temperature on the shaker. After another washing step, the diluted streptavidin-PE solution was added for 30 min. The read of the plate was performed within 60 minutes.

### Statistical analysis

Statistical analysis was performed using Graph Pad Prism, v5 (Graph Pad Software, San Diego, CA, USA) and SPSS, v25 (IBM SPSS Software, Chicago, IL, USA). Figures were created with Graph Pad Prism, v5. Data are presented as scatter dots or bars with information about the mean and SEM. Each figure legend contains the relevant statistical information: the n, total number of participants, the significance p-value, and the statistical test used. All data were checked for both normality and variance using the Kolmogorov-Smirnov test. The parametric data were analyzed using the unpaired t-test and one-way ANOVA with *Post-hoc* Tukey’s Multiple Comparison test. The majority of the data were non-parametric and the statistical tests applied were: Mann-Whitney test (the non-parametric counterpart to unpaired t-test), and Kruskal-Wallis with Dunn’s Multiple Comparison test (the non-parametric counterpart to one-way ANOVA). Spearman’s correlation coefficients (R) were used to assess positive or negative associations between measured variables. R values between 0.2-0.39 were treated as weak, between 0.4-0.59 as moderate, and between 0.6-0.79 as very strong correlation factors. The linear regression analyses related to main **Figures 2** and 5 were performed using Graph Pad Prism v5 in order to identify the predictive value of factor X (independent variable plotted on the X axis) on factor Y (dependent variable plotted on the Y axis). Each linear regression graph shows the best-fit line with the 95% confidence band. The coefficient of determination R-squared (R^2^) was used as a goodness-of-fit measure and the F-test to determine the level of significance. The linear regression models related to **Figure 7** and **Table 2** were generated using SPSS v25 for predicting the antibody response (the dependent variable) based on the serum concentrations of various independent variables (negative and positive factors). R^2^ and adjusted R^2^ were used as goodness-of-fit measures and ANOVA test was applied for assessing the statistical significance for the proposed predictive models. The dependent variable may be determined based on the expression of multiple independent variables (predictors, p): *B_0_* (constant) + *B_1_X_1_* + *B_2_X_2_* +… + *B_p_Xp.* The logistic regression analyses used for predicting the previous-infection status were performed using SPSS, v25. More precisely, receiver operating characteristic (ROC) curves were generated to compare the sensitivity (*s_n_*) versus specificity (*s_p_*) across a range of possible cut-off values, and the area under those curves (AUC) was used as a measure of test performance. The optimal cut-off values were determined by identifying the minimum distance from the ROC curve to the upper left corner point (where *s_n_* =1 and *s_p_* =1). The distance between this point (*s_n_* =1, *s_p_* =1) and any point on the ROC curve is $d=\;\sqrt{{\left(1-s_{n}\right)}^{2}+{\left(1-s_{p}\right)}^{2}}$ which was calculated for each observed cut-off value in order to locate the minimum. The results for AUC are reported as area, standard error of the area (S.E.), 95% confidence interval of the area and *P* value (testing the null hypothesis that AUC=0.5). For negative predictors, the smaller values of the test result variables indicate stronger evidence for a positive actual state (previous infection), while for positive predictors, the larger values of the test variables suggest the previous infection status. The *P* values less than 0.05 was considered statistically significant.

## Results

### RBD-specific antibody responses following BNT162b2 dual vaccination are weaker in people over 60 years old

**Figure 1:**
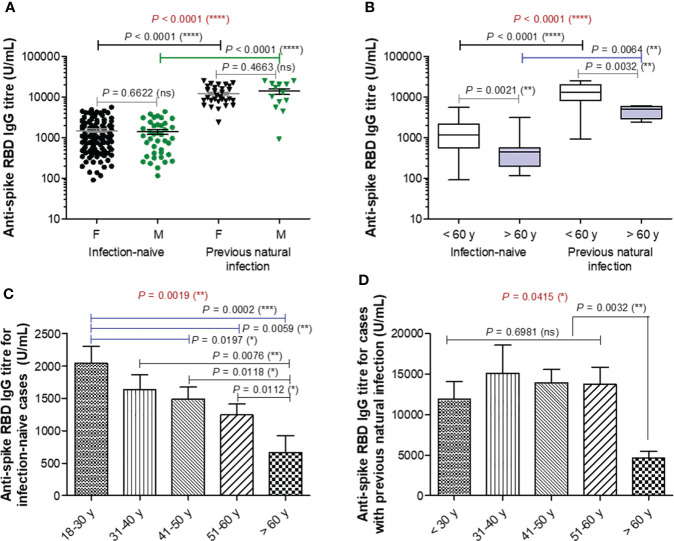
Spike RBD-specific IgG titre after two doses of BNT162b2 (BioNTech-Pfizer) vaccination. **(A)** Spike RBD-specific antibody titre after two doses of COVID-19 vaccination in both groups of subjects stratified by gender: infection-naïve (109 females, 40 males) and previously infected (30 females, 13 males) cases. The black and grey lines indicate the mean ± SEM (****P< 0.0001, ns, not significant; two-tailed Mann Whitney and Kruskal-Wallis followed by Dunn’s Multiple Comparison tests). **(B)** Spike RBD-specific antibody titre after two doses of COVID-19 vaccination in both groups of subjects stratified by age: infection-naïve (n = 149) and previously infected (n=33) cases. The black and grey lines indicate the mean ± SEM (****P< 0.0001, **P< 0.01, ns, not significant; two-tailed Mann Whitney and Kruskal-Wallis followed by Dunn’s Multiple Comparison tests). **(C, D)** Spike RBD-specific antibody titre in **(C)** infection-naïve and **(D)** infection-primed individuals stratified by age. Bars indicate the mean ± SEM (****P< 0.0001, ***P < 0.001, **P< 0.01, *P< 0.05, ns, not significant; two-tailed Mann Whitney and Kruskal-Wallis followed by Dunn’s Multiple Comparison tests).

Blood samples were collected from 192 participants (149 [77.6%] were infection-naïve, while 43 [22.4%] declared previous natural infection) who had completed the second vaccination with BNT162b2 (participants’ characteristics are displayed in **Supplementary Table 1**). Blood samples were collected at a median of 74 (IQR 48-91) and 75 (IQR 43-91) days after the second vaccination for the infection-naïve and previously infected (also called infection-primed) groups of subjects, respectively. The previously infected group showed a significant 8.67-fold increase in the spike RBD-specific IgG titre compared to the infection-naïve group (mean values for the two groups: 12674 U/mL for infection-primed *vs.* 1462 for infection-naïve, *P*< 0.0001). Each group was further subdivided according to gender, and the female to male ratios were 2.7 and 2.3 for the infection-naïve and previously infected groups, respectively. Both females and males showed similar antibody titres in each main group (**Figure 1A**). When stratified by age, the subgroup of subjects older than 60 years showed significant reduction in the magnitude of the antibody response in both infection-naïve (2.24-fold difference between< 60 years old and > 60 years old) and – primed (2.92-fold difference between< 60 years old and > 60 years old) categories. Therefore, the fold increase in the previously-infected subjects was 8.82 for people< 60 years old, and to a less extent of 6.76 for people > 60 years old (**Figure 1B**). Interestingly, the subjects showed a consistent trend of antibody titre decrease with age within the infection-naïve group. For instance, while individuals younger than 30 years had a mean titre value of 2042 U/mL, the population over 60 years had 3-times less, 668 U/mL (*P* = 0.0002) – **Figure 1C**. By contrast, within the previously infected group, the antibody titres were similar among the individuals younger than 60 years with a general mean of 13501 U/mL, however significantly higher than those seen in the subjects aged 60 years or older characterized by a titre mean of 4610 U/mL (*P* = 0.0032) **Figure 1D**. Our data clearly showed that across both main groups, infection-naïve and previously infected, there was a significant reduction in the antibody response following vaccination in older individuals.

### RBD-specific antibody responses following BNT162b2 dual vaccination do not depend on vitamin D levels

**Figure 2:**
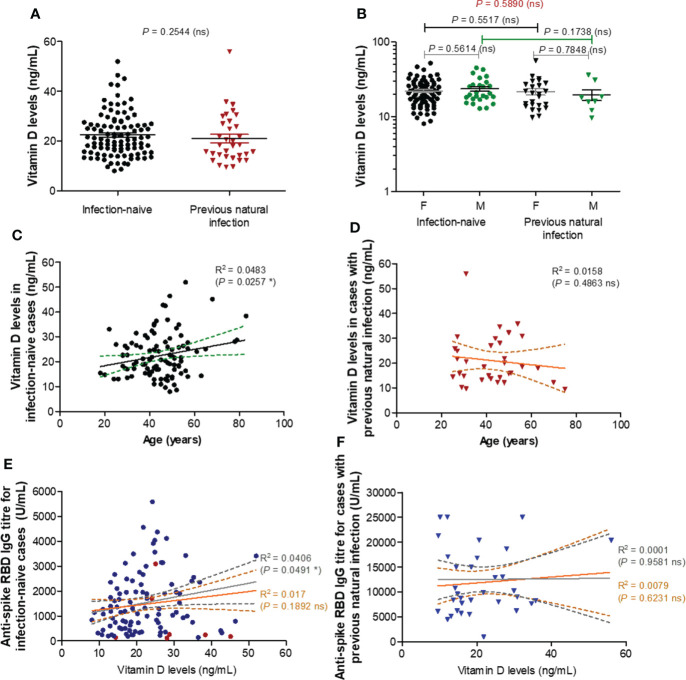
Vitamin D levels moderately correlate with RBD specific IgG antibody titres in individuals under 60 years. **(A)** Vitamin D levels in the serum collected from both groups of subjects: infection-naïve (n = 103) and previously infected (n = 33) cases. The black and red lines indicate the mean ± SEM (ns, not significant; two-tailed Mann test). **(B)** Vitamin D levels according to gender in infection-naïve (77 females, 26 males) and previously infected (25 females, 8 males) cases. The black and grey lines indicate the mean ± SEM (ns, not significant; two-tailed Mann test). **(C)** The effect of age on vitamin D levels in infection-naïve subjects (R, correlation coefficient; *P< 0.05; F test). **(D)** The effect of age on vitamin D levels in infection-primed subjects (R, correlation coefficient; ns, not significant; F test). **(E)** The effect of vitamin D levels on the RBD-specific antibody response in infection-naïve subjects (R, correlation coefficient; *P< 0.05, ns, not significant; F test). The grey lines correspond to the group of individuals younger than 60 years, while the orange lines indicate the entire infection-naïve group. **(F)** The effect of vitamin D levels on the RBD-specific antibody response in infection-primed subjects (R, correlation coefficient; ns, not significant; F test). The grey lines correspond to the group of individuals younger than 60 years, while the orange lines indicate the entire infection-primed group.

To further elucidate the reason of this almost 2-3 times reduction in the spike RBD-specific antibody titre observe after vaccination in older subjects, we first investigated the role of vitamin D (25-OH) levels, if any, in controlling the antibody response. Surprisingly, there were no significant differences in the vitamin D serum levels between the two groups (*P* = 0.2544, **Figure 2A**) infection-naïve (mean value of 22.44 ng/mL) and infection-primed (mean value of 21.01 ng/mL), or between females and males (**Figure 2B**). Additionally, within the infection-naive group, the vitamin D levels revealed a weak correlation with age (R = 0.220 [95% CI 0.028 to 0.397], *P* = 0.0257), as only one subject over 60 years had lower value than the general mean (**Figure 2C**). However, this correlation was not seen in the previously-infected group (**Figure 2D**). Further, no associations between vitamin D levels and antibody responses were identified in our study groups (**Figures 2E, F**), except a week, but significant correlation observed only among the infection-naïve individuals younger than 60 years (R = 0.201 [95% CI 0.001-0.386], *P* = 0.0491, **Figure 2E**). These data clearly suggest that vitamin D levels do not play an important role in assessing the antibody response after BNT162b2 dual vaccination in both infection-naïve and previously-infected individuals.

### Serum levels of pro-inflammatory biomarkers (MCP-1, CRP, factor D, PAI-1) decrease after previous infection and BNT162b2 vaccination compared to vaccination alone

**Figure 3:**
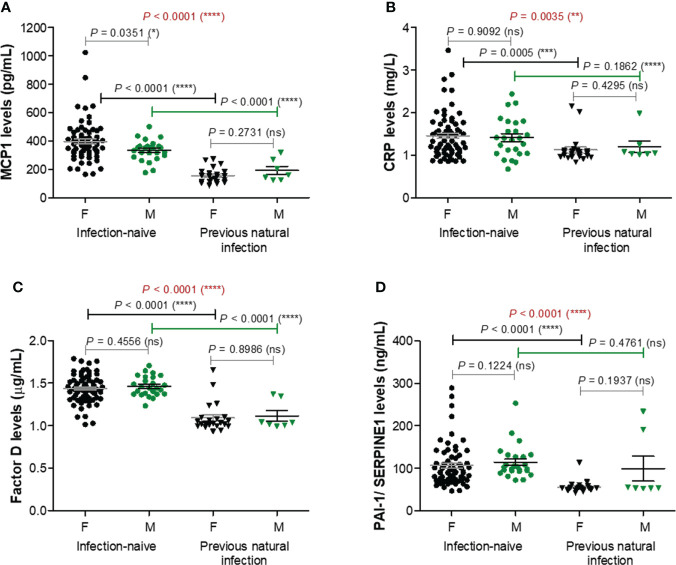
Plasma profile of pro-inflammatory molecules following BNT162b2 vaccination in previously infected or naïve individuals. **(A)** MCP-1 levels, **(B)** CRP levels, **(C)** Factor D levels, and **(D)** PAI-1/SERPINE1 levels in the serum samples collected from infection-naïve (n = 92, 67 females, 25 males) and previously infected (n = 30, 23 females, 7 males) cases. The black and grey lines indicate the mean ± SEM (****P< 0.0001, ***P< 0.001, **P< 0.01, *P< 0.05, ns, not significant; two-tailed Mann Whitney and Kruskal-Wallis followed by Dunn’s Multiple Comparison tests). F, females; M, males.

As adipose tissue was recently shown to influence both the COVID-19 outcome and antibody generation targeting spike protein of SARS-CoV2 after vaccination or natural infection, we next explored the serum levels of ten well described adipose tissue-related factors: MCP-1/CCL2, CRP, factor D, PAI-1/SERPINE1, interleukins 6 (IL-6) and 10 (IL-10), adiponectin, leptin, resistin, and TNF-α. Spike-specific antibody titres for cases included in this analysis are shown in **Supplementary Figure 1**. The first four listed factors (MCP-1, CRP, factor D, PAI-1) showed significant lower levels in the serum of previously infected individuals compared to the infection-naïve people, with no significant differences according to gender. For MCP-1 levels within the infection-naïve group, the mean values were 397.5 pg/mL (95% CI 362.9-432.2) for females and 334.8 pg/mL (95% CI 305.1-364.5) for males. By comparison, the corresponding values within the infection-primed group showed an overall 2.3-fold significant decrease (*P*< 0.0001), of 156.4 pg/mL (95% CI 133.0-179.9) and 192.6 pg/mL (95% CI 122.0-263.3), respectively – **Figure 3A**. Interestingly, the case with the highest MCP-1 serum levels (1024.96 pg/mL) also associated relatively higher values for the other pro-inflammatory molecules CRP (2.20 mg/L), factor D (1.66 μg/mL), PAI-1 (166.58 ng/mL), and antibody titre (3416 U/mL). Regarding the CRP serum levels, the differences were less pronounced, the overall fold-change being only 1.27 (*P* = 0.0035, **Figure 3B**). For Factor D and PAI-1 the values were reduced by 1.32 and respectively 1.64 times in the infection-primed group when compared to the infection-naïve individuals (*P*< 0.0001, **Figures 3C, D**). As the values’ distribution of those four biomarkers within the previously-infected group was clearly skewed to the right, we next investigated what those cases with extreme unexpected higher values had in common. Interestingly, those cases were the oldest in the group, being over 60 years. As shown in **Supplementary Figures 2A−D**, the general reduction of the four biomarkers’ values seen in the infection-primed group was only visible within the people younger than 60 years (P< 0.0001), while the older group (over 60 years) did not show any change compared to the counterpart infection-naïve cases. Also, while within the infection-naïve group there were no significant differences among the two subgroups of individuals< 60 years old and > 60 years old, within the infection-primed group the differences were indeed striking (*P*< 0.01, **Supplementary Figures 3A−D**). As those four biomarkers showed a consistent decrease in the infection-primed individuals (especially younger than 60 years), we called them as negative factors/determinants for previous infection.

### Serum levels of adipokines (adiponectin, leptin, IL-6, IL-10) increase after previous infection and BNT162b2 vaccination compared to vaccination alone

**Figure 4:**
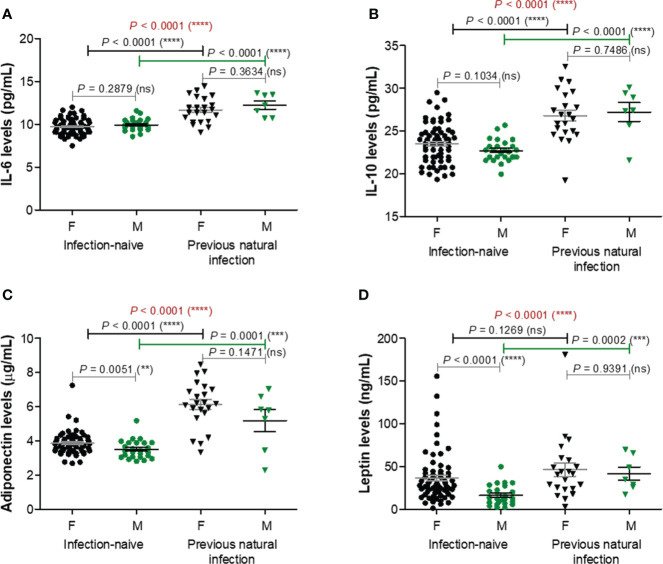
Plasma profile of adipokines following BNT162b2 vaccination in previously infected or naïve individuals. **(A)** IL-6 levels, **(B)** IL-10 levels, **(C)** Adiponectin levels, and **(D)** Leptin levels in the serum samples collected from infection-naïve (n = 92, 67 females, 25 males) and previously infected (n = 30, 23 females, 7 males) cases. The black and grey lines indicate the mean ± SEM (****P< 0.0001, ns, not significant; two-tailed Mann Whitney and Kruskal-Wallis followed by Dunn’s Multiple Comparison tests). F, females; M, males.

Among the next studied molecules, IL-6 and IL-10 concentrations were significantly increased (*P*< 0.0001) in the infection-primed group (11.82 pg/mL [95% CI 11.28-12.36] and 26.90 pg/mL [95% CI 25.79-28.01]), compared with the infection-naïve one (9.79 pg/mL [95% CI 9.61-9.97] and 23.30 pg/mL [95% CI 22.86-23.73]); however, IL-6 and IL-10 concentrations were not influenced by gender – **Figures 4A, B**. Adipokines like adiponectin and leptin also increased in the infection-primed group. For instance, adiponectin levels were significantly higher by 1.57 times (P< 0.0001) in the infection-primed group (5.90 μg/mL [95% CI 5.34-6.47]) when compared to the infection-naïve cases (3.76 μg/mL [95% CI 3.63-3.89]). The adiponectin levels were slightly influenced by gender only in the infection-naïve group: females had higher values than males (3.86 μg/mL [95% CI 3.70-4.02]) *vs.* 3.50 μg/mL [955 CI 3.28-3.73], *P* = 0.0051, **Figure 4C**). Leptin serum levels were also higher in the previously-infected individuals (45.31 ng/mL [95% CI 32.54-58.09]) compared to infection-naïve subjects (31.11 ng/mL [95% CI 25.55-36.67]) and higher in females compared to males within the last group (36.59 ng/mL [95% CI 29.56-43.63] *vs.* 16.43 ng/mL [95% CI 11.61-21.25, P< 0.0001], **Figure 4D**). Considering the higher dispersion of values seen among the previously-infected individuals, we next stratified the cases by age. Similarly to the case of the first four analyzed biomarkers, the overall increase seen in IL-6, IL-10, adiponectin or leptin serum levels were due to their increase only in the younger group (< 60 years old). The older infection-primed group did not show any change in those adipokines concentrations compared to the counterpart infection-naïve cases. Therefore, significant differences were detected among younger and older individuals within the infection-primed group for IL-6 (12.01 pg/mL [95% CI 11.42-12.60] for< 60 years old *vs.* 10.65 pg/mL [95% CI 9.53-11.78] for > 60 years old, *P* = 0.0387) and adiponectin (6.32 μg/mL [95% CI 5.86-6.77] for< 60 years old *vs.* 3.32 μg/mL [95% CI 2.07-4.56] for > 60 years old) concentrations. As those four biomarkers increased in the infection-primed individuals (especially younger than 60 years), we called them as positive factors/determinants for previous infection.

TNF-α and resistin did not show any changes among different studied groups of subjects, except for TNF-α which was only influenced by gender irrespective of the infection status (**Supplementary Figure 4**). Overall, these data suggest that the lower vaccination-induced antibody response seen in the older individuals who were previously exposed to natural infection is followed by no-change in adipokine biomarkers. Interestingly, in the case of previously-infected younger individuals, who showed at least 8.8-fold increase of antibody response compared to their counterparts from the infection-naïve group associated a significant reduction in the concentration of proinflammatory biomarkers (negative factors: MCP-1, CRP, factor D, PAI-1), concomitant with an increase in the serum levels of other adipokines, such as adiponectin, leptin, IL-6, and IL-10. These observations are important as they might also explain the discrepancies observed in the fold increase of anti-RBD antibody responses caused by previous infection and vaccination compared to vaccination alone in individuals younger than 60 years (8.82, *P* = 0.0021) and older than 60 years (6.76, *P* = 0.0032) – **Figure 1B**.

### Serum levels of adiponectin strongly correlate with RBD-specific antibody responses and negatively with age following BNT162b2 dual vaccination in previously-infected individuals

**Figure 5:**
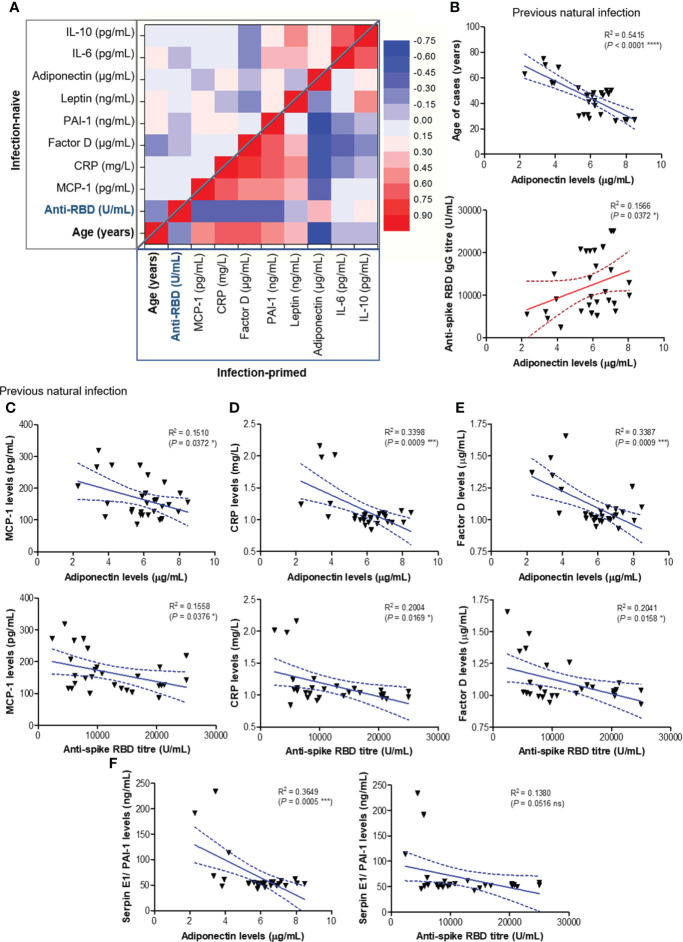
Regression statistics describing the relationship between age, antibody response and various adipokines within the two main study groups: infection-naïve and infection-primed. Correlation coefficients (R) and statistical significances were computed for each pair of variables. **(A)** Heat map of R coefficients: left –upper corner corresponds to infection naïve cases, while the bottom-right corner corresponds to infection-primed cases. Linear regression analysis for **(B)** adiponectin levels and age (top) or adiponectin and RBD-specific antibody titre (bottom); **(C)** MCP-1 and adiponectin levels (top) or MCP-1 levels and antibody titre (bottom); **(D)** CRP and adiponectin levels (top) or CRP levels and antibody titre (bottom); **(E)** factor D and adiponectin levels (top) or factor D levels and antibody titre (bottom); **(F)** PAI-1/SERPINE1 and adiponectin levels (top) or PAI-1/SERPINE1 levels and antibody titre (bottom) in previously infected individuals. Data are presented as scatter plots with best-fit lines and 95% confidence bands (****P< 0.0001, ***P< 0.001, *P<0.05, ns, not significant; Spearman test).

To further assess the association between age or spike RBD-specific antibody responses with various adipokines concentrations, we generated a regression statistical analysis depicted in **Figure 5A**. In the infection-naïve group, the strongest negative relationships were observed between age and Factor D levels or between age and anti-RBD titre, as expected. Interestingly, Factor D levels revealed a moderate negative dependency on the concentrations of distinct positive factors, while weak or moderate correlations were also noticed between any combinations of positive factors (top right corner in **Figure 5A**). However, strong and very strong relationships were revealed between age, antibody response, positive and negative factors within the infection-primed group. While age positively correlated with negative factors and inversely associated with positive factors, the antibody titres mirrored those effects (positive correlations with positive factors and inverse correlations with negative factors). Interestingly, the strongest relationship resulted from the inverse association of age with adiponectin levels (R = -0.736 [95% CI -0.868 to -0.506], *P*< 0.0001, **Figure 5B**). As expected, a significant dependency of antibody response on adiponectin levels was confirmed (*P* = 0.0372, **Figure 5B**). All four negative factors (MCP-1, CRP, factor D, PAI-1) inversely correlated with adiponectin levels in previously-infected individuals following dual vaccination (**Figures 5C−F**). Among them, CRP (*P* = 0.0009), factor D (*P* = 0.0009) and PAI-1 (*P* = 0.0005) levels showed the highest inverse association with adiponectin concentrations. Importantly, while for adiponectin concentrations higher than 4.2 μg/mL (corresponding to individuals younger than 56 years), the variation of CRP (mean 1.021 mg/L [95% CI 0.99-1.05]), factor D (mean 1.03 μg/mL [95% CI 1.00-1.06]), PAI-1 (mean 52.63 ng/mL [95% CI 50.93-54.33]) levels were relatively reduced, adiponectin concentration lower than 5.3 μg/mL (corresponding to individuals older than 56 years) associated an increase of 57.8% in CRP levels (mean 1.61 mg/L [95% CI 1.10-2.12], *P* = 0.0014, **Figure 5D**), 32.0% in factor D levels (mean 1.36 μg/mL [95% CI 1.14-1.57], *P* = 0.0009, **Figure 5E**), and 126.3% in PAI-1 levels (mean 119.1 ng/mL [95% CI 57.53-199.7], *P* = 0.0123, **Figure 5F**). As expected, all four negative factors negatively correlated also with the spike RBD-specific antibody titres (**Figures 5C−F**). Overall, these data clearly indicate that, among all investigated adipokines, adiponectin serum levels best correlate with age (negative association) and antibody titre (positive correlation). Our results also suggest that the reduced adiponectin levels seen in older individuals are associated with and might explain the lack of reduction in the concentration of negative factors (MCP-1, CRP, factor D, PAI-1) after dual vaccination, reduction which was noticed only in people younger than 60 years.

### Increased expression of proinflammatory biomarkers with age is associated with adiponectin reduction

**Figure 6:**
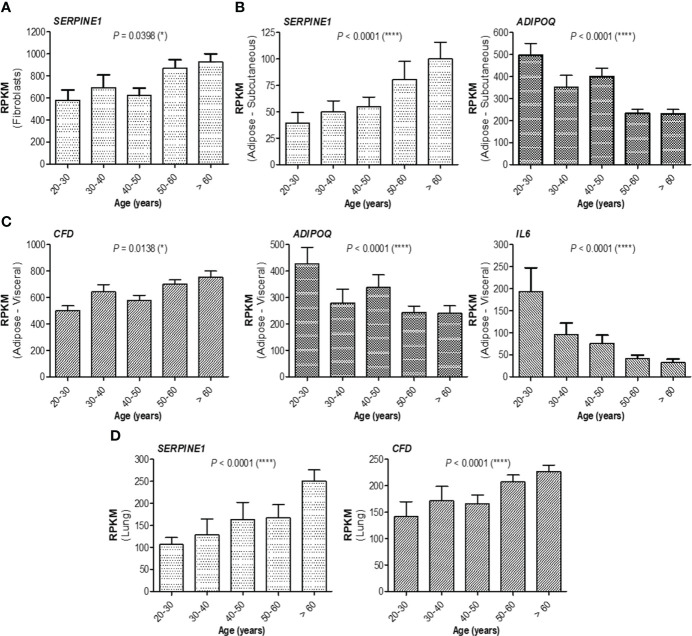
mRNA expression of adipokines collected from GTEx database. **(A)** mRNA expression of *SERPINE1* in fibroblasts, **(B)** mRNA expression of *SERPINE1* and *ADIPOQ* in subcutaneous adipose tissue, **(C)** mRNA expression of *CFD, ADIPOQ*, and *IL6* in visceral adipose tissue, and **(D)** mRNA expression of *SERPINE1* and *CFD* in the lung. The bars indicate the mean ± SEM (****P< 0.0001, *P< 0.05; Kruskal-Wallis test).

At this point, combining those observations with the previous ones, where the antibody response in individuals over 60 years was much weaker and the magnitude of antibody increase in the infection-primed group was less than in the case of people younger than 60 years, we concluded that the best factors identified to associate with age, showing either inverse (for adiponectin, IL-6, IL-10) or positive (for PAI-1, factor D, CRP) correlation are key in explaining the findings in older subjects, and probably, those relationships are normally present in the general population unexposed to infection or vaccine. Therefore, to validate this hypothesis, we investigated the expression of these factors using the normal tissue data from GTEx database generated before the pandemic. For instance, in fibroblasts, *SERPINE1* mRNA expression (encoding for PAI-1) significantly increased with age (*P* = 0.0398, **Figure 6A**), as well as in the subcutaneous adipose tissue (*P*< 0.0001), accompanied by a decrease in *ADIPOQ* mRNA expression (encoding for adiponectin, *P<* 0.0001, **Figure 6B**). Consistently, in visceral adipose tissue, *CFD* expression (encoding for factor D) increased, while *ADIPOQ* and *IL6* expression diminished with age (**Figure 6C**). Interestingly, in lung we only identified *SERPINE1* and *CFD* mRNA expression to change with age (**Figure 6D**), as expected by increasing. All the other factors not shown in **Figure 6** did not significantly correlate with age. These data suggest that the baseline levels of positive factors decrease with age, while the baseline levels for negative factors (proinflammatory biomarkers) increase with age, thus making the older individuals to have an important delay (or blockage) in the dynamic response of key adipokines and consequently a lower antibody titre outcome.

### Serum levels of MCP-1, factor D, adiponectin and IL-6 are good predictors for RBD-specific antibody responses within individuals younger than 60 years

**Figure 7:**
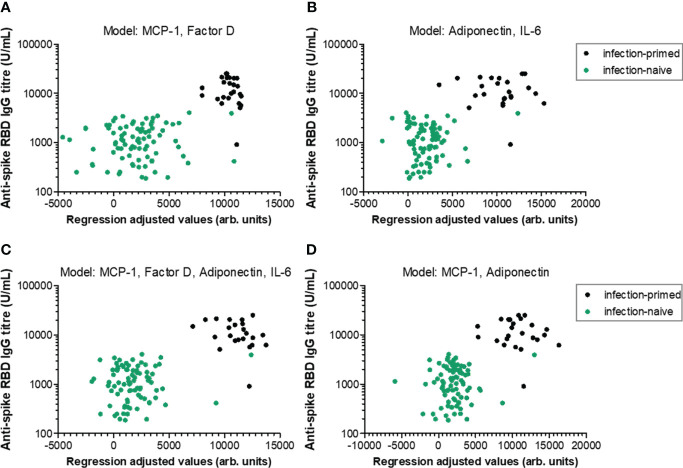
Linear regression models generated to predict the antibody response within individuals younger than 60 years. Antibody responses in relation to regression-adjusted predicted values generated by associating **(A)** negative factors such as MCP-1 and factor D, **(B)** positive factors such as adiponectin and IL-6, or **(C)** combined factors such as MCP-1, factor D, adiponectin, IL-6, or **(D)** MCP-1 and adiponectin. Infection-primed cases are depicted as black dots, while the infection-naïve cases are shown in green.

We next aimed to define regression-based prediction models for antibody responses following dual BNT162b2 vaccination, starting from the serum concentrations of negative and positive factors (**Table 1** and **Supplementary Tables 2−4** for extended statistical evaluation of regression coefficients). The first proposed model (Model 1-1) investigated the prediction strength of here-in identified negative factors: MCP-1, CRP, factor D and PAI-1. Among those, only MCP-1 and Factor D yielded a significant contribution to the model, but only within the age group younger than 60 years (adj. R^2^ = 0.504, *P*< 0.001; **Figure 7A**; **Table 1** and **Supplementary Table 2**). Similarly, among positive predictors (Model 1-2: leptin, adiponectin, IL-6, IL-10), only adiponectin and IL-6 serum concentration proved to significantly contribute to the model within individuals< 60 years old (adj. R^2^ = 0.500, *P*< 0.001; **Figure 7B**; **Table 1** and **Supplementary Table 3**). The combined effect of both positive and negative factors provided only a modest improvement to the model (< 60 years: adj. R^2^ = 0.569, *P*< 0.001; **Figures 7C, D**; **Table 1** and **Supplementary Table 4**), suggesting that the serum concentration of either negative or positive factors is sufficient to predict the RBD-specific antibody responses within subjects younger than 60 years irrespective of the previous natural infection status.

**Table 1:** Statistical evaluation for the indicated prediction models.

Model (linear regression)	R^2^	Adjusted R^2^	*P* value (ANOVA)
***Negative factors (Model 1-1)***			
MCP-1_CRP_Factor D_PAI-1 < 60 years > 60 years	0.508 0.522 0.249	0.490 0.503 -0.251	< 0.001 < 0.001 0.740
MCP1_Factor D < 60 years > 60 years	0.500 0.513 0.153	0.491 0.504 -0.058	< 0.001 < 0.001 0.513
***Positive factors (Model 1-2)***			
Leptin_Adiponectin_IL-6_IL-10 < 60 years > 60 years	0.486 0.510 0.373	0.468 0.491 -0.045	< 0.001 < 0.001 0.523
Adiponectin_IL-6 < 60 years > 60 years	0.485 0.509 0.304	0.476 0.500 0.130	< 0.001 < 0.001 0.235
***Combined factors (Model 1-3)***			
MCP1_Factor D_ Adiponectin_IL-6 < 60 years > 60 years	0.572 0.585 0.602	0.557 0.569 0.336	< 0.001 < 0.001 0.177
MCP-1_Adiponectin < 60 years > 60 years	0.497 0.513 0.319	0.488 0.504 0.149	< 0.001 < 0.001 0.215

### Serum concentrations of MCP-1, factor D, and PAI-1 are negative determinants for previous-infection

**Figure 8:**
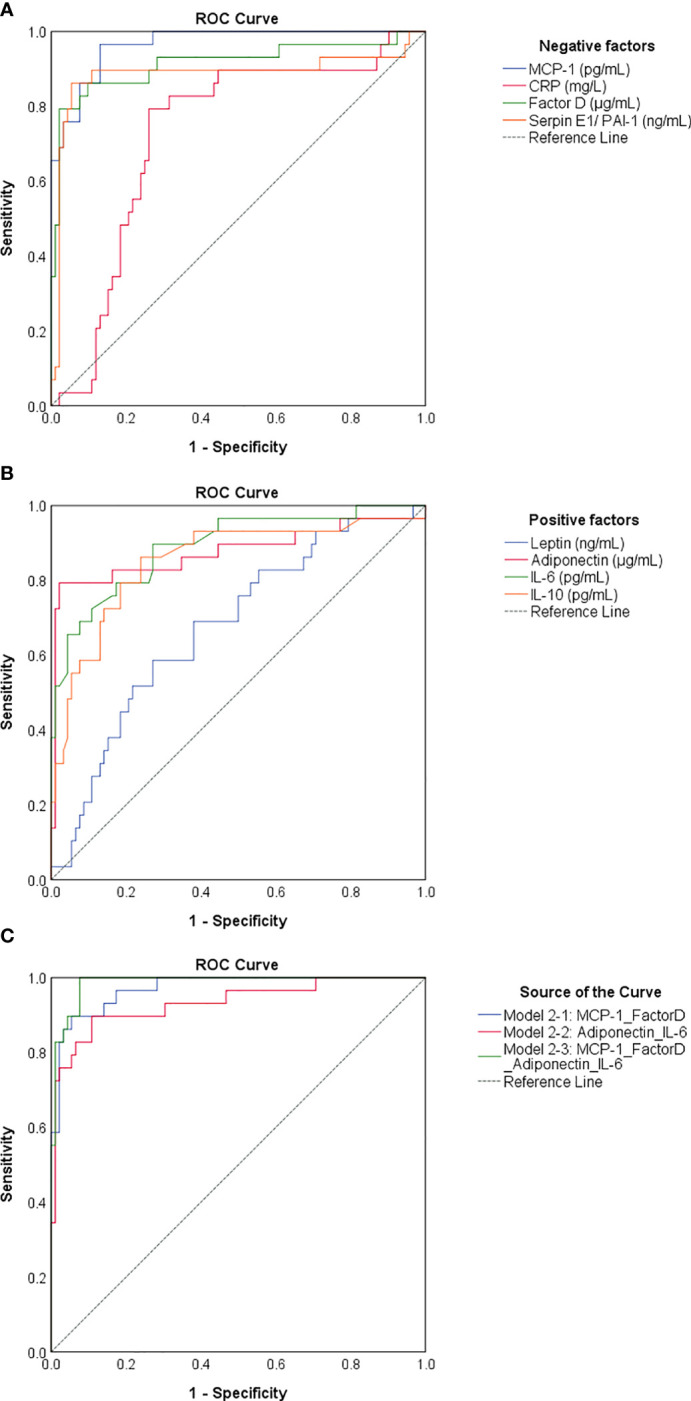
ROC curves generated for negative and positive determinants of previous natural infection. ROC curves for **(A)** negative and **(B)** positive determinants. **(C)** ROC curves related to various associations of biomarkers. Model 2-1 comprises the values of MCP-1 and Factor D, model 2-2 comprises the values of adiponectin and IL-6, and model 2-3 comprises the biomarkers included in model 2-1 and 2-2. The AUC values between 0.8-0.9 define an excellent discrimination, while the AUC values > 0.9 denote an outstanding capacity of prediction.

As the identified negative and positive factors changed significantly in cases with previous infection and vaccination compared to vaccination alone, we next wondered which of them would best predict the infection-primed status in the general population. For this we generated a Receiver Operating Characteristic (ROC) analysis for each negative and positive factor – **Table 2**. All four negative factors proved to be significant negative determinants/predictors for previous infection (P< 0.0001), however only MCP-1, factor D and PAI-1 yielded area under curve (AUC) values > 0.8 (MCP-1: 0.966 [95% CI 0.938-0.995]; factor D: 0.914 [95% CI 0.838-0.990]; PAI-1: 0.885 [95% CI 0.787-0.984], **Table 2**; **Figure 8A**). For MCP-1 serum levels, a cut-off value of 272.76 pg/mL, was associated with 0.97 sensitivity and 0.87 specificity, and for factor D levels, a cut-off value of 1.26 μg/mL, was associated with 0.86 sensitivity and 0.90 specificity. However, at a cut-off value of 62.52 ng/mL for PAI-1, the assay achieved the highest specificity of 0.95 (sensitivity 0.86) (**Table 2**). All positive factors also proved to be good determinants/ predictors for previous infection (**Table 2**; **Figure 8B**). The highest AUC values were achieved for IL-6 (0.890 [95% CI 0.818-0.963], *P*< 0.0001) and adiponectin (0.876 [95% CI 0.779-0.972], *P*< 0.0001). For IL-6, at a cut-off of 10.67 pg/mL the sensitivity was 0.79 and the specificity was 0.82, while for adiponectin, a cut-off value of 5.26 μg/mL yielded a sensitivity of 0.79 and a high specificity of 0.98. To further validate these results, we next conducted a logistic regression analysis for defining various association models. As such, the association of either both selected negative predictors (model 2-1: MCP-1_FactorD) or both selected positive predictors (model 2-2: Adiponectin_IL-6) yielded to an outstanding discrimination for the subjects who had previous natural infection (**Figure 8C**). As expected, the model 3-1 which comprised the first 2 models had the highest AUC value of 0.987 [95% CI 0.972-1.000] (**Supplementary Table 5**). Since age and gender are the most profound confounders in adipokine studies, we included them as covariates in our binary logistic regression models. Interestingly, age and gender provided a light improvement only to the adiponectin-based models (AUC varied from the initial value of 0.876 [95% CI 0.779-0.972] to 0.920 [95% CI 0.852-0.988], **Supplementary Figure 5** and **Supplementary Table 6**). Overall, these models achieved high predictivity, similar to the one indicated by antibody titres *per se* (AUC 0.981 [95% CI 0.954-1.000]) for an optimal cut-off value of 4424 U/mL (sensitivity 0.95 and specificity 0.97, **Supplementary Figure 6**). Therefore, these data suggest that infection status may be indeed determined by the expression of negative factors (which is reduced in the infection-primed group) and positive factors (which is increased in the infection-primed group).

**Table 2:** Statistical evaluation of biomarkers for previous infection prediction.

Analyte	AUC	S.E.	*P* value	Confidence interval (CI)	Cut-off value	Sensitivity	Specificity
***Negative factors***							
MCP-1 (pg/mL)	0.966	0.014	< 0.001	0.938-0.995	272. 76	0.966	0.870
CRP (mg/L)	0.725	0.053	< 0.001	0.621-0.828	1.11	0.793	0.739
Factor D (μg/mL)	0.914	0.039	< 0.001	0.838-0.990	1.26	0.862	0.902
PAI-1 (ng/mL)	0.885	0.050	< 0.001	0.787-0.984	62.52	0.862	0.946
***Positive factors***							
IL-6 (pg/mL)	0.890	0.037	< 0.001	0.818-0.963	10.67	0.793	0.826
IL-10 (pg/mL)	0.852	0.046	< 0.001	0.761-0.942	24.50	0.862	0.761
Leptin (ng/mL)	0.677	0.056	0.004	0.567-0.786	28.77	0.690	0.620
Adiponectin (μg/mL)	0.876	0.049	< 0.001	0.779-0.972	5.26	0.793	0.978

## Discussions

Here we have identified the antibody responses following dual BNT162b2 vaccination in infection-naïve or previously-infected individuals. Despite no significant change of spike RBD-specific antibody titre by gender, we found a significant lower antibody response in individuals over 60 years, effect more pronounced within the previously-infected group of participants. A recent study performed in the United Kingdom (UK) reported an important impairment in the immune response among older people (> 65 years old) only in the infection-naïve group, while similar high antibody titres were noticed irrespective of age in the infection-primed group (4). These discrepancies seen between our results and the data from the UK population are very interesting and may be due to several reasons. First, the UK study included in the analysis only blood samples collected at 6 days after second vaccination, while in our study the samples were collected at a median of 75 [IQR 47-91] days. Second, the vaccination scheme differed between the two studies: in our research the second vaccination was performed at 21 days after the first dose as imposed by our national regulations, while in the UK, due to limited availability, the second dose’s administration was delayed up to 3 months by the UK authorities (18). Nevertheless, any differences in the genetic background between UK and Romanian nationals may also contribute to this outcome. Importantly, other studies also confirmed a higher magnitude of antibody response in previously-infected compared to naïve individuals after BNT162b2 vaccination (19–21).

To understand the differences in humoral immune responses between younger and older people, we next investigated the role of vitamin D. This was important, as vitamin D deficiency (< 20 ng/mL) was largely reported to influence the severity of COVID-19 (22, 23), as it is known to be associated with an increase in inflammatory cytokines (24, 25) and thrombotic episodes (26, 27). However, recent studies performed on nationwide cohorts were not able to identify any association between vitamin D deficiency and hospitalization or mortality due to COVID-19, suggesting that there is still insufficient scientific evidence for the role of vitamin D levels in COVID-19 infection (28, 29). Other studies investigated the dependency of antibody response following vaccination with SARS-CoV-2 vaccines on vitamin D concentration and found no significant association (10, 11). In our work, we only revealed a weak, but significant correlation between vitamin D serum levels and antibody titres following BNT162b2 vaccination in infection-naive individuals younger than 60 years. Surprisingly, most of the individuals over 60 years included in our research had higher vitamin D concentrations than the general mean of 22.10 ng/mL [95% CI 20.56-23.63].

Little is known about the relationship between adipokines and humoral immune responses following vaccination with SARS-CoV-2 vaccines. Adipokines are mainly produced by adipose tissues (subcutaneous and visceral) and are known to influence the immune system in multiple ways. For instance, adiponectin has anti-inflammatory actions as it suppresses the synthesis of pro-inflammatory cytokines (TNF-α, IL-6, and MCP-1) by monocytes/macrophages (30–32), while inducing the production of anti-inflammatory mediators like IL-1 receptor antagonist and IL-10 (33). On the other hand, leptin, a pro-inflammatory adipokine induces the production of TNF-α, IL-6, and IL-10 by human B cells in *in-vitro* studies (34). There are also cross-regulations, as IL-6 acts on adipose tissue to promote leptin secretion (35). PAI-1 is another adipokine with a key role in suppressing intravascular and tissue fibrinolysis, and such, high levels are associated with deregulated vascular coagulation and endothelial dysfunction (36). Elevated PAI-1 circulating concentration also causes insulin resistance contributing to the generation of a metabolic syndrome, and may, in turn, be influenced by multiple cytokines, growth factors and hormones (36, 37). Interestingly, it has been recently shown that PAI-1 also promotes a respiratory innate antiviral immunity (38, 39). In our study we have identified several differences in the circulating levels of multiple adipose tissue-related factors between infection-naïve and infection-primed individuals. Of note, previously-infected subjects had higher levels of adiponectin and leptin which were correlated with higher IL-6 and IL-10, potentially reflecting polarization towards Th2 responses which rather boost the humoral immune responses (antibody production) and impede the cellular immunity. As such, IL-10 is a key cytokine involved in B cell activation, proliferation, antibody production, and class-switch towards IgG_1_ and IgG_3_ (40–42). Apart from the role of adiponectin in regulating the synthesis of IL6 and IL-10, it was recently shown to be able to directly induce B cell proliferation and differentiation by activating key signaling pathways involving the phosphoinositide-3-kinase (PI3K)/Akt1 and signal transducer and activator of transcription 3 (STAT3) (43). Additionally, among circulating lymphocytes, adiponectin receptors are mainly expressed on B cells, and in response to adiponectin stimulation, B cells secrete a peptide PEPITEM which inhibits T cell trafficking to inflamed tissues, thus diminishing inflammation. Of interest, the expression of adiponectin receptors on B cells wanes with age, contributing further to immune-senescence (44). However, these hypotheses require validation in future in-depth studies. Furthermore, increased adiponectin levels were also associated with a reduction in several pro-inflammatory molecules: MCP-1, factor D, CRP and PAI-1, indicating a potential suppression of innate immunity. In the UK study, the MCP-1 levels did not change, and only TNF-α and CXCL10 increased in the infection-primed subjects at 6 days post-vaccination, suggesting a potential polarization towards a Th1 phenotype (4). Interestingly, our observed changes were only present in individuals younger than 60 years. The older individuals might develop suboptimal immune responses as they seemed not to be responsive to those molecular changes triggered by previous infection and vaccination, thus explaining the relatively lower raise in antibody response observed in this category compared to the younger group. This observation might be important, also because it is known that adipokine dysfunction is another factor associated with aging that may induce various metabolic changes by promoting a low-grade inflammation (45, 46). It has been shown that circulating concentrations of adiponectin either increase (45, 47) or do not change with age (48). Surprisingly, the mRNA expression for adiponectin was reduced in the adipose tissue from the subjects included in the GTEx database. This observation might imply the fact that, despite a general lower synthesis of adiponectin by adipose tissues, there is less clearance due to adiponectin resistance. Obviously, one of the important questions still remains to be addressed in future research in the context of COVID-19 infection: is it desirable an intense Th2 response with humoral immunity or a Th1 phenotype that augments the cellular immunity? Among all studied biomarkers, circulating MCP-1 and factor D acted as negative factors, while adiponectin and IL-6 as positive factors in predicting the magnitude of antibody response following dual BNT162b2 vaccination within individuals younger than 60 years. Interestingly, for determining the previous infection status, circulating MCP-1, factor D and PAI-1 proved to be excellent negative predictors. By contrast, adiponectin and IL-6 serum levels positively associated with the previous exposure status.

Our study has some limitations. Firstly, we did not have access to the information regarding the exact time or severity of the infection in the previously-infected individuals. However, the vaccination was performed at least 90 days post-disease as recommended by local authorities, and as the samples were collected at 2-3 months after dual BNT162b2 vaccination (performed at 21 days interval), we can argue that the RBD-specific antibody responses produced by vaccination did not confound with the antibody levels produced by infection. Secondly, we did not store the information about the body mass index or additional comorbidities at the time of blood sample collection, information which is expected to impact on our analysis. Still, we had access to the retrospective hospital database, and identified that around 9% of included subjects were recorded with obesity, figure similar to the one reported recently (in 2019) for obesity prevalence in our country by the Eurostat data (49). Of note, the previously-infected individuals included in our research are those that survived to the primary exposure. Additionally, some subgroups included a limited number of individuals, as this study was designed as a retrospective analysis of prospectively collected samples.

Our data are of importance as they reveal the humoral immune responses following standard dual vaccination at a 2-3 months interval (median of 75 days). Firstly, the previously-infected individuals had much higher antibody titres than the infection-naïve people, indicating that repeated vaccination might be less needed for them. Additionally, older subjects had suboptimal antibody responses, suggesting that new vaccine designs might be required to offer a better protection for this category of individuals more susceptible to develop severe forms of COVID-19. Still, it might be necessary to even consider adjusting the vaccination scheme (only 21 days or more)? in order to achieve the desired protective immune response.

## Data availability statement

The original contributions presented in the study are included in the article/**Supplementary Material**. Further inquiries can be directed to the corresponding author.

## Ethics statement

The studies involving human participants were reviewed and approved by Institutional Ethics Committee - St. Spiridon County Clinical Emergency Hospital of Iasi. The patients/participants provided their written informed consent to participate in this study.

## Author contributions

MP-T, CGT, and PC conceptualized the study. MP-T, EA, AM, and CGT collected the samples. All authors participated in performing the experiments. MP-T performed the statistical analysis and prepared the draft which was reviewed and commented by all authors.

## Funding

We are grateful for funding from a grant of the Romanian Ministry of Research, Innovation and Digitization, CNCS/CCCDI-UEFISCDI, project number PN-III-P1-1.1-PD-2019-0733 (MP-T and PC), within PNCDI-III, and POC/448/1/1/127606 CENEMED project no. 367/390043/2021.

## Acknowledgments

The authors would like to thank all co-workers from the Laboratory of Immunology at St. Spiridon County Clinical Emergency Hospital of Iasi for their support during the research time and to express their deepest gratitude to all participants in this study.

## Conflict of interest

The authors declare that the research was conducted in the absence of any commercial or financial relationships that could be construed as a potential conflict of interest.

## Publisher’s note

All claims expressed in this article are solely those of the authors and do not necessarily represent those of their affiliated organizations, or those of the publisher, the editors and the reviewers. Any product that may be evaluated in this article, or claim that may be made by its manufacturer, is not guaranteed or endorsed by the publisher.
