# Supplementary material for: Adipokines, and not vitamin D, associate with antibody immune responses following dual BNT162b2 vaccination within individuals younger than 60 years
Source: Front Immunol. 2022 Sep 2;13:1000006. doi: 10.3389/fimmu.2022.1000006 (PMC9481237; doi:10.3389/fimmu.2022.1000006)
Supplement: Supplementary file 1 [file DataSheet_1.pdf]

Adipokines, and not vitamin D, associate with antibody immune responses following dual BNT162b2 vaccination within individuals younger than 60 years

Supplementary materials contain:

Supplementary Figures 1-6

Supplementary Tables 1-6

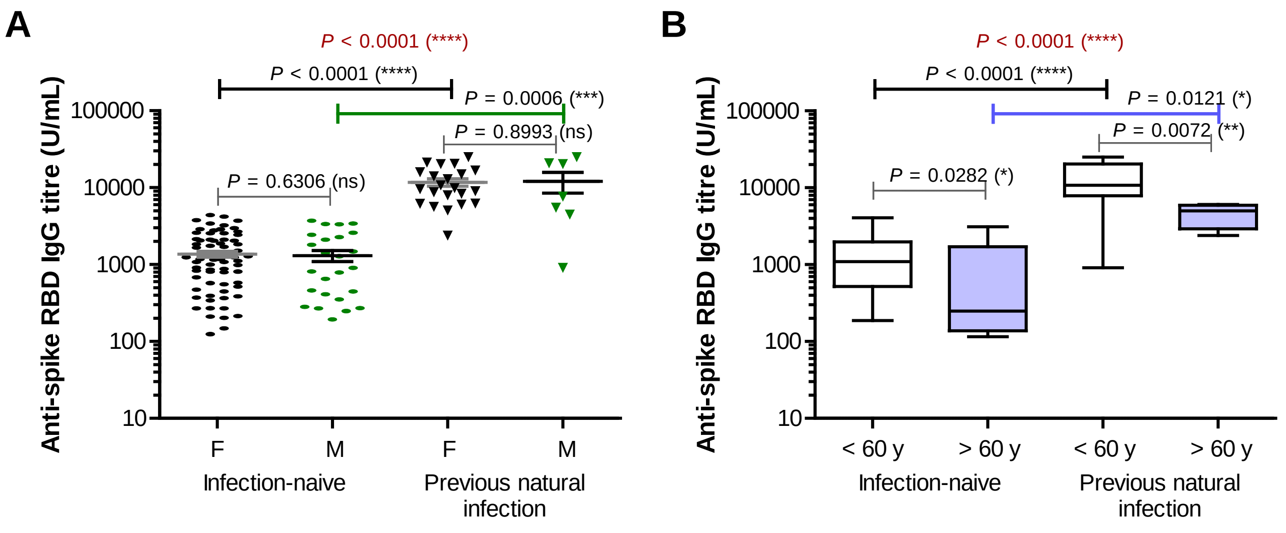

Supplementary Figure 1. Spike RBD-specific IgG titre after two doses of BNT162b2 (BioNTech-Pfizer) vaccination for the 122 participants in the adipokines' assessment study. (A) Spike RBD-specific antibody titre after two doses of COVID-19 vaccination in both groups of subjects stratified by gender: infection-naïve (68 females, 25 males) and previously infected (22 females, 7 males) cases. The black and grey lines indicate the mean ± SEM (*****P*< 0.0001, ****P*< 0.001, ns, not significant; two-tailed Mann Whitney and Kruskal-Wallis followed by Dunn's Multiple Comparison tests). **(B)** Spike RBD-specific antibody titre after two doses of COVID-19 vaccination in both groups of subjects stratified by age: infection-naïve (n = 92) and previously infected (n=30) cases. The black and grey lines indicate the mean ± SEM (*****P*< 0.0001, ***P*< 0.01, **P*< 0.05, ns, not significant; two-tailed Mann Whitney and Kruskal-Wallis followed by Dunn's Multiple Comparison tests).

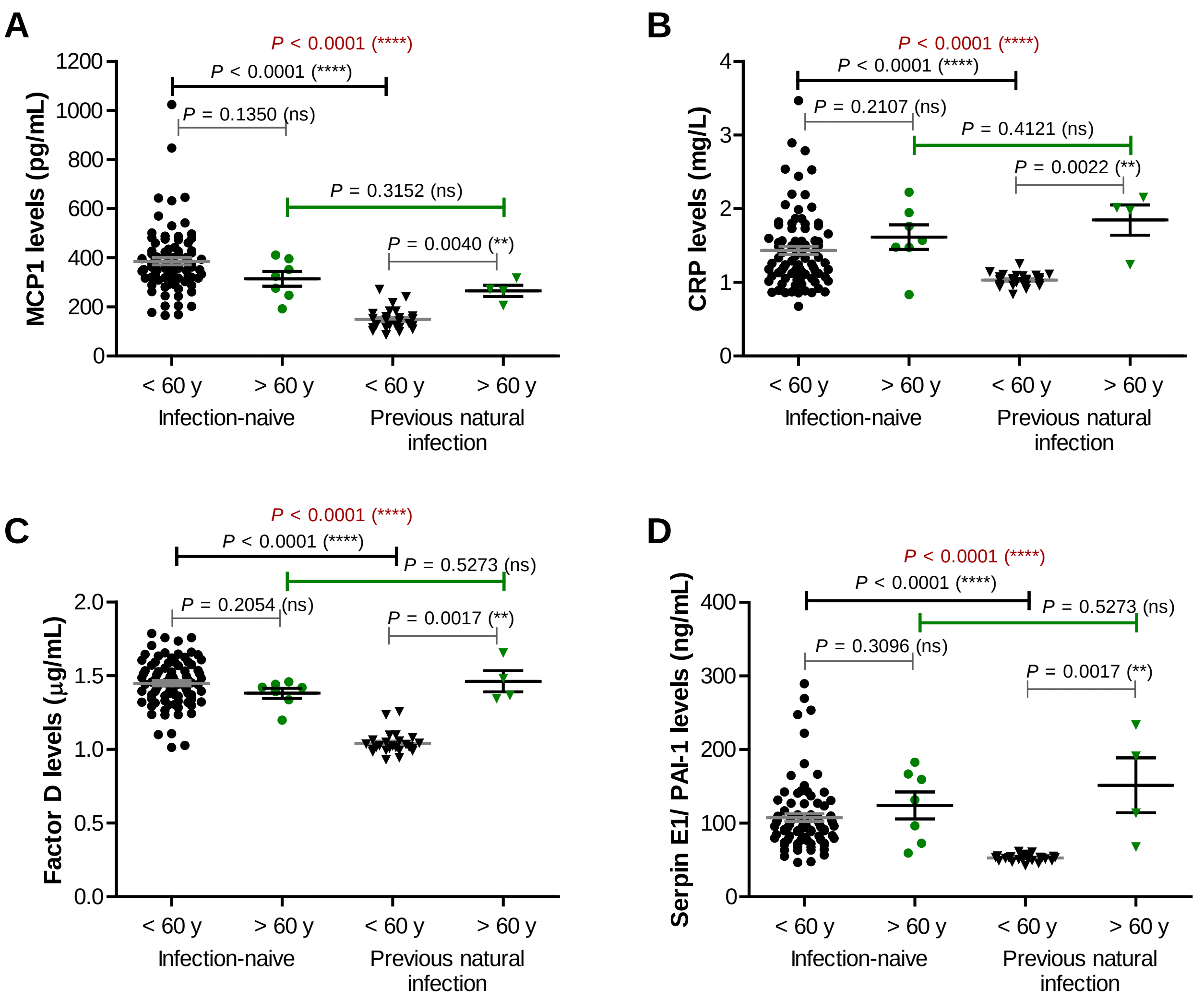

Supplementary Figure 2. Plasma profile of pro-inflammatory molecules following BNT162b2 vaccination in previously infected or naïve individuals.

(A) MCP-1 levels, **(B)** CRP levels, **(C)** Factor D levels, and **(D)** PAI-1 levels in the serum samples collected from infection-naïve (n = 92) and previously infected (n = 30) cases. The black and grey lines indicate the mean ± SEM (****$P < 0.0001$, **$P < 0.01$, ns, not significant; two-tailed Mann Whitney and Kruskal-Wallis followed by Dunn’s Multiple Comparison tests).

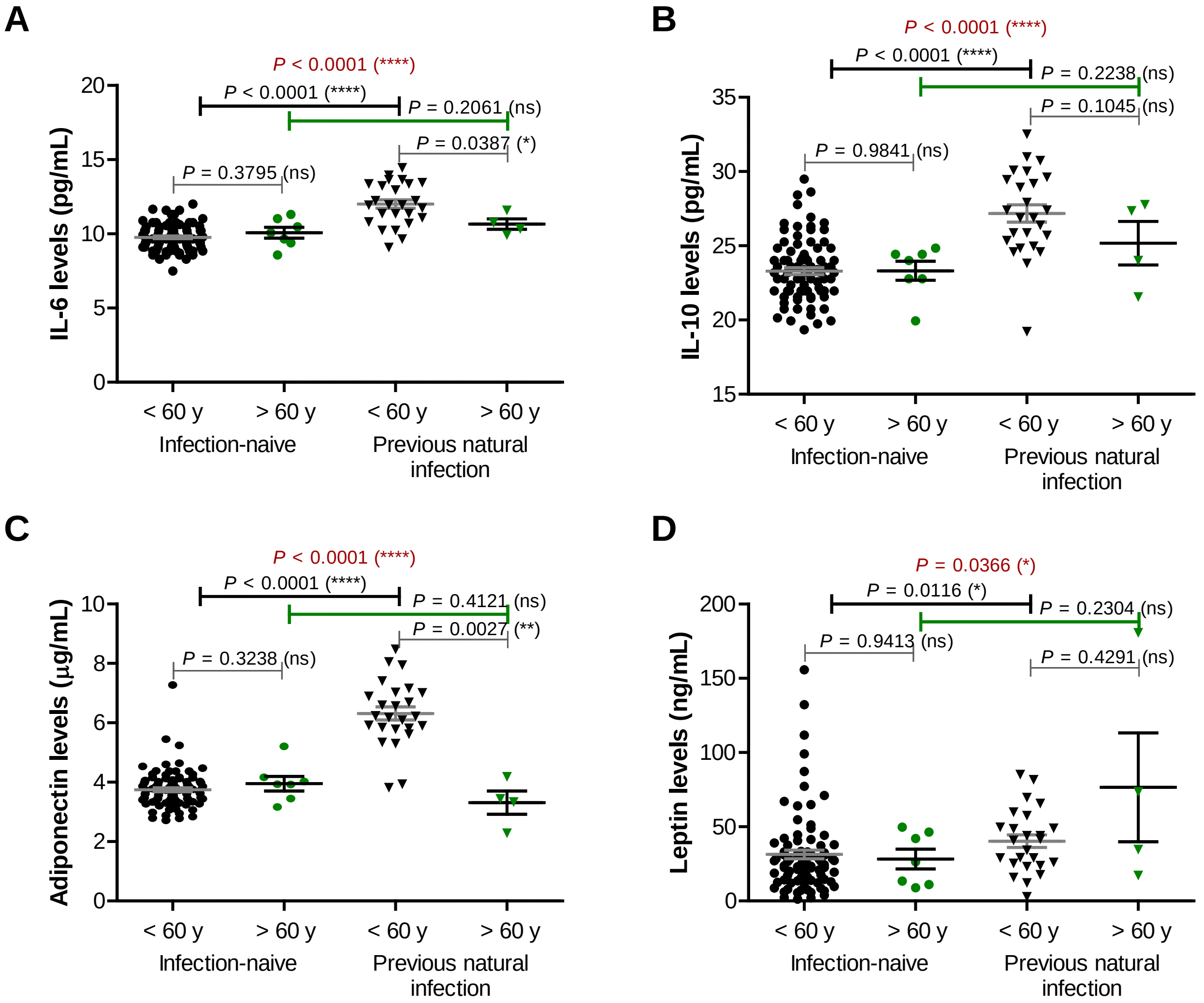

Supplementary Figure 3. Plasma profile of adipokines following BNT162b2 vaccination in previously infected or naïve individuals.

(A) IL-6 levels, **(B)** IL-10 levels, **(C)** Adiponectin levels, and **(D)** Leptin levels in the serum samples collected from infection-naïve (n = 92) and previously infected (n = 30) cases. The black and grey lines indicate the mean ± SEM (*****P*< 0.0001, ***P*< 0.01, **P*< 0.05, ns, not significant; two-tailed Mann Whitney and Kruskal-Wallis followed by Dunn's Multiple Comparison tests).

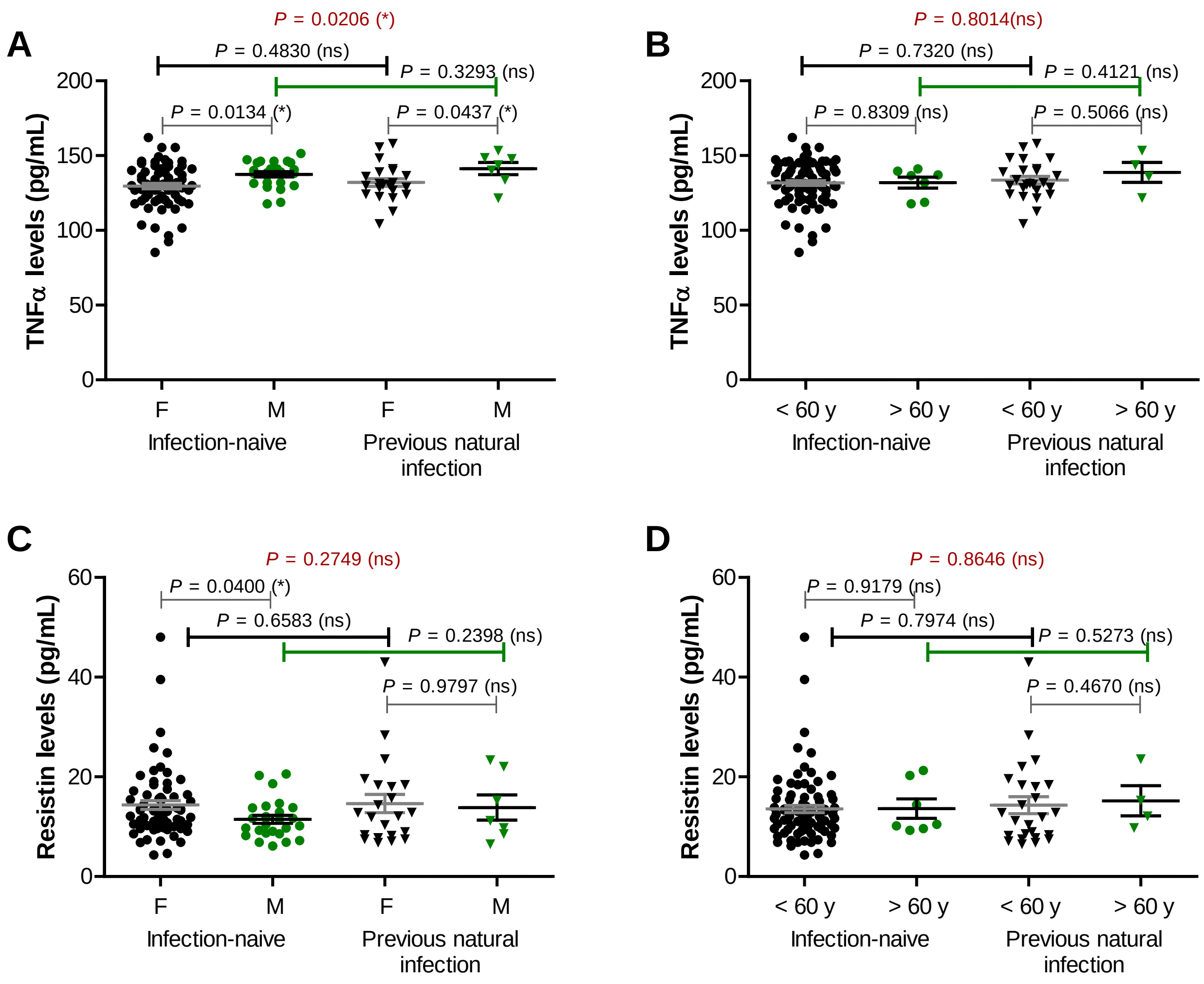

Supplementary Figure 4. Plasma profile of TNF-α and resistin following BNT162b2 vaccination in previously infected or naïve individuals.

Serum levels of TNF-α in individuals stratified by **(A)** gender or **(B)** age, and serum levels of resistin in individuals stratified **(C)** gender, or **(D)** age categorized as infection-naïve (n = 92) and previously infected (n = 30). The black and grey lines indicate the mean ± SEM (*$P < 0.05$, ns, not significant; two-tailed Mann Whitney and Kruskal-Wallis followed by Dunn's Multiple Comparison tests).

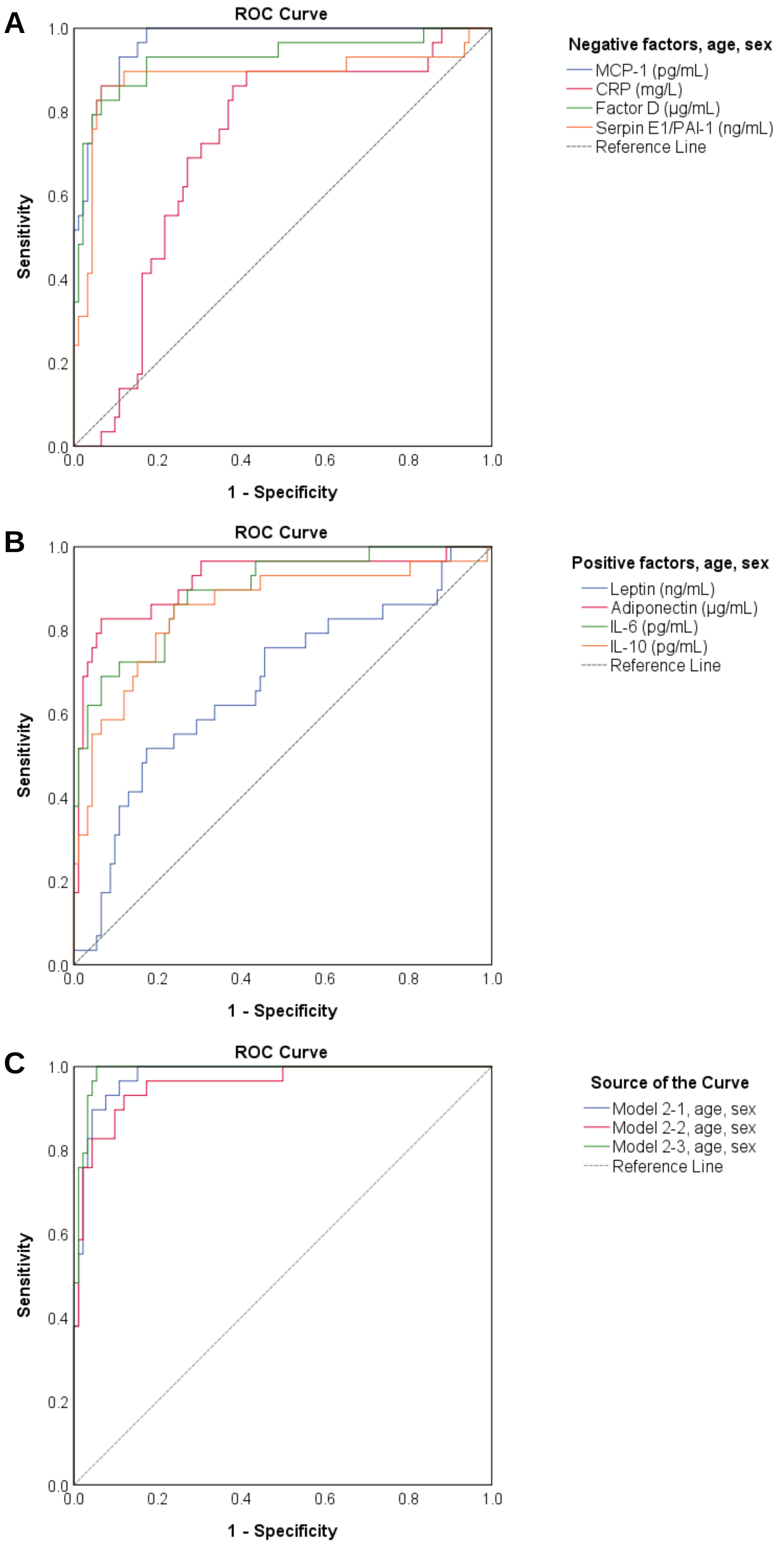

Supplementary Figure 5. ROC curves generated for negative and positive predictors of previous natural infection and adjusted for age and sex.

ROC curves for models including age, sex and (A) negative or (B) positive predictors. (C) ROC curves related to various associations of biomarkers with age and sex. Model 2-1 comprises the values of MCP-1 and Factor D, model 2-2 comprises the values of adiponectin and IL-6, and model 2-3 comprises the biomarkers included in model 2-1 and 2-2.

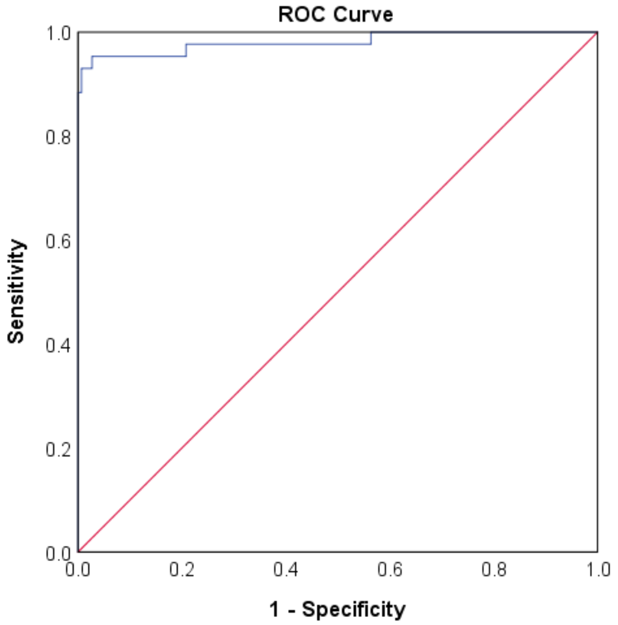

Analyte	AUC	S.E.	*P* value	Confidence interval (CI)	Cut-off value	Sensitivity	Specificity
Anti-spike RBD IgG titre (U/mL)	0.981	0.014	< 0.001	0.954-1.000	4424.00	0.953	0.973

Supplementary Figure 6. ROC curve generated for RBD-specific antibody response as a positive predictor for previous natural infection.
The AUC and optimal cut-off values are listed in the table bellow. An AUC value > 0.9 denotes an outstanding capacity of prediction.

Supplementary Table 1. General characteristics of participants for assessing spike-specific antibody responses.

Characteristic	Infection-naïve group (n = 149)	Infection-primed group (n = 43)	All cases (n = 192)
**Age, years**			
< 60	137 (92%)	39 (91%)	176 (92%)
Median age [IQR]	45 [37-51]	45 [30-50]	
Mean age (SEM)	43.28 (0.81)	41.13 (1.64)	*P* = 0.2394 (ns)
> 60	12 (8%)	4 (9%)	16 (8%)
Median age [IQR]	64.5 [62-67]	69 [64.25-73.25]	
Mean age (SEM)	68.42 (2.28)	69.00 (2.48)	*P* = 0.5836 (ns)
**Sex**			
Female	109 (73%)	30 (70%)	139 (72%)
Median age [IQR]	48 [38-52]	46 [28-51.25]	
Mean age (SEM)	45.44 (1.01)	42.33 (2.44)	*P* = 0.1789 (ns)
Male	40 (27%)	13 (30%)	53 (28%)
Median age [IQR]	44.5 [36.25-53]	46 [40.5-52.5]	
Mean age (SEM)	44.95 (2.27)	46.92 (3.13)	*P* = 0.6540 (ns)

Supplementary Table 2. Coefficients of linear regression models used to predict the antibody response based on the serum levels of negative factors.

All cases		(Constant)	MCP-1 (pg/mL)	CRP (mg/L)	Factor D (µg/mL)	PAI-1 (ng/mL)
Unstandardized Coefficients	B	25587.564	-14.304	-102.287	-11374.383	-2.646
	S.E.	2429.591	3.448	84.751	2120.244	9.293
Standardized Coefficients	Beta		-0.338	-0.087	-0.431	-0.023
t		10.532	-4.148	-1.207	-5.365	-0.285
P value		0.000	0.000	0.230	0.000	0.776

< 60 years		(Constant)	MCP-1 (pg/mL)	CRP (mg/L)	Factor D (µg/mL)	PAI-1 (ng/mL)
Unstandardized Coefficients	B	26091.874	-14.305	-116.817	-11588.250	-1.781
	S.E.	2537.614	3.760	90.726	2229.588	10.809
Standardized Coefficients	Beta		-0.337	-0.094	-0.438	-0.014
t		10.282	-3.805	-1.288	-5.197	-0.165
P value		0.000	0.000	0.201	0.000	0.869

> 60 years		(Constant)	MCP-1 (pg/mL)	CRP (mg/L)	Factor D (µg/mL)	PAI-1 (ng/mL)
Unstandardized Coefficients	B	7178.042	-16.734	193.062	-2733.193	4.117
	S.E.	14623.402	14.112	279.005	10248.377	15.162
Standardized Coefficients	Beta		-0.519	0.356	-0.133	0.102
t		0.491	-1.186	0.692	-0.267	0.272
P value		0.641	0.281	0.515	0.799	0.795

All cases		(Constant)	MCP-1 (pg/mL)	Factor D (µg/mL)
Unstandardized Coefficients	B	25255.188	-14.615	-12276.791
	S.E.	2400.041	3.214	2005.044
Standardized Coefficients	Beta		-0.346	-0.465
t		10.523	-4.547	-6.123
P value		0.000	0.000	0.000

< 60 years		(Constant)	MCP-1 (pg/mL)	Factor D (µg/mL)
Unstandardized Coefficients	B	25612.855	-14.603	-12438.969
	S.E.	2499.681	3.405	2125.478
Standardized Coefficients	Beta		-0.344	-0.470
t		10.246	-4.288	-5.852
P value		0.000	0.000	0.000

> 60 years		(Constant)	MCP-1 (pg/mL)	Factor D (µg/mL)
Unstandardized Coefficients	B	2309.121	-11.108	2248.696
	S.E.	11367.453	11.026	7020.132
Standardized Coefficients	Beta		-0.344	0.109
t		0.203	-1.007	0.320
P value		0.844	0.343	0.757

Supplementary Table 3. Coefficients of linear regression models used to predict the antibody response based on the serum levels of positive factors.

**All cases**		**(Constant)**	**Leptin (ng/mL)**	**Adiponectin (µg/mL)**	**IL-6 (pg/mL)**	**IL-10 (pg/mL)**
Unstandardized Coefficients	B	-17549.067	1.632	2081.069	1050.210	63.311
	S.E.	3761.503	16.166	357.963	483.088	268.802
Standardized Coefficients	Beta		0.009	0.492	0.259	0.032
t		-4.665	0.101	5.814	2.174	0.236
P value		0.000	0.920	0.000	0.032	0.814

**< 60 years**		**(Constant)**	**Leptin (ng/mL)**	**Adiponectin (µg/mL)**	**IL-6 (pg/mL)**	**IL-10 (pg/mL)**
Unstandardized Coefficients	B	-17863.508	1.275	2187.155	996.402	82.399
	S.E.	4000.845	19.155	380.852	520.031	286.239
Standardized Coefficients	Beta		0.006	0.511	0.244	0.041
t		-4.465	0.067	5.743	1.916	0.288
P value		0.000	0.947	0.000	0.058	0.774

**> 60 years**		**(Constant)**	**Leptin (ng/mL)**	**Adiponectin (µg/mL)**	**IL-6 (pg/mL)**	**IL-10 (pg/mL)**
Unstandardized Coefficients	B	7697.285	18.591	-1666.724	719.936	-315.469
	S.E.	10625.786	22.924	1031.089	1073.092	548.839
Standardized Coefficients	Beta		0.395	-0.530	0.277	-0.313
t		0.724	0.811	-1.616	0.671	-0.575
P value		0.496	0.448	0.157	0.527	0.586

**All cases**		**(Constant)**	**Adiponectin (µg/mL)**	**IL-6 (pg/mL)**
Unstandardized Coefficients	B	-17159.212	2088.483	1163.501
	S.E.	2819.890	338.842	324.942
Standardized Coefficients	Beta		0.494	0.287
t		-6.085	6.164	3.581
P value		0.000	0.000	0.001

**< 60 years**		**(Constant)**	**Adiponectin (µg/mL)**	**IL-6 (pg/mL)**
Unstandardized Coefficients	B	-17341.881	2197.455	1139.281
	S.E.	2924.779	358.598	342.346
Standardized Coefficients	Beta		0.514	0.279
t		-5.929	6.128	3.328
P value		0.000	0.000	0.001

**> 60 years**		**(Constant)**	**Adiponectin (µg/mL)**	**IL-6 (pg/mL)**
Unstandardized Coefficients	B	5402.274	-1705.841	304.203
	S.E.	8516.710	927.839	765.946
Standardized Coefficients	Beta		-0.543	0.117
t		0.634	-1.839	0.397
P value		0.544	0.103	0.702

Supplementary Table 4. Coefficients of linear regression models used to predict the antibody response based on the serum levels of both negative and positive factors.

**All cases**		**(Constant)**	**MCP-1 (pg/mL)**	**Factor D (μg/mL)**	**Adiponectin (μg/mL)**	**IL-6 (pg/mL)**
Unstandardized Coefficients	B	1954.906	-10.023	-5888.145	1134.304	795.934
	S.E.	5459.023	3.072	2217.833	372.148	318.476
Standardized Coefficients	Beta		-0.244	-0.230	0.268	0.196
t		0.358	-3.262	-2.655	3.048	2.499
P value		0.721	0.001	0.009	0.003	0.014

**< 60 years**		**(Constant)**	**MCP-1 (pg/mL)**	**Factor D (μg/mL)**	**Adiponectin (μg/mL)**	**IL-6 (pg/mL)**
Unstandardized Coefficients	B	1544.362	-9.510	-5864.360	1191.830	800.979
	S.E.	5800.788	3.291	2358.199	407.465	337.250
Standardized Coefficients	Beta		-0.231	-0.228	0.278	0.196
t		0.266	-2.889	-2.487	2.925	2.375
P value		0.791	0.005	0.014	0.004	0.019

**> 60 years**		**(Constant)**	**MCP-1 (pg/mL)**	**Factor D (μg/mL)**	**Adiponectin (μg/mL)**	**IL-6 (pg/mL)**
Unstandardized Coefficients	B	-14131.587	2.928	13724.660	-2739.646	609.693
	S.E.	12147.600	12.072	7246.619	1167.164	740.435
Standardized Coefficients	Beta		0.091	0.668	-0.871	0.235
t		-1.163	0.243	1.894	-2.347	0.823
P value		0.289	0.816	0.107	0.057	0.442

**All cases**		**(Constant)**	**MCP-1 (g/mL)**	**Adiponectin (μg/mL)**
Unstandardized Coefficients	B	-499.771	-13.909	2066.505
	S.E.	2253.581	3.292	341.536
Standardized Coefficients	Beta		-0.329	0.471
t		-0.222	-4.225	6.051
P value		0.825	0.000	0.000

**< 60 years**		**(Constant)**	**MCP-1 (pg/mL)**	**Adiponectin (μg/mL)**
Unstandardized Coefficients	B	-988.702	-13.362	2158.543
	S.E.	2482.262	3.525	369.100
Standardized Coefficients	Beta		-0.315	0.486
t		-0.398	-3.791	5.848
P value		0.691	0.000	0.000

**> 60 years**		**(Constant)**	**MCP-1 (pg/mL)**	**Adiponectin (μg/mL)**
Unstandardized Coefficients	B	9377.293	-6.052	-1450.485
	S.E.	3790.961	10.331	1006.383
Standardized Coefficients	Beta		-0.188	-0.461
t		2.474	-0.586	-1.441
P value		0.038	0.574	0.187

Supplementary Table 5. Statistical evaluation of various prediction models.

Analyte	AUC	S.E.	*P* value	Confidence interval (CI)
**Model 2-1** (MCP-1_Factor D)	0.971	0.014	< 0.001	0.944-0.998
**Model 2-2** (Adiponectin_IL-6)	0.933	0.031	< 0.001	0.872-0.993
**Model 2-3** (MCP-1_Factor D_ Adiponectin_IL-6)	0.987	0.08	< 0.001	0.972-1.000

Supplementary Table 6. Statistical evaluation of biomarkers for previous infection prediction, adjusted for age and sex.

Model	AUC	S.E.	*P* value	Confidence interval (CI)
Negative factors, age, sex				
MCP-1 (pg/mL)	0.969	0.013	< 0.001	0.943-0.994
CRP (mg/L)	0.713	0.052	0.001	0.610-0.815
Factor D (μg/mL)	0.927	0.034	< 0.001	0.860-0.993
PAI-1 (ng/mL)	0.883	0.049	< 0.001	0.788-0.979
Positive factors, age, sex				
IL-6 (pg/mL)	0.892	0.035	< 0.001	0.824-0.961
IL-10 (pg/mL)	0.850	0.046	< 0.001	0.759-0.941
Leptin (ng/mL)	0.670	0.060	0.006	0.552-0.788
Adiponectin (μg/mL)	0.920	0.035	< 0.001	0.852-0.988
Models, age, sex				
Model 2-1	0.977	0.011	< 0.001	0.955-0.999
Model 2-2	0.957	0.020	< 0.001	0.917-0.997
Model 2-3	0.988	0.007	< 0.001	0.974-1.000
